# Supplementary material for: Host Cell Rap1b mediates cAMP-dependent invasion by Trypanosoma cruzi
Source: PLoS Negl Trop Dis. 2023 Mar 10;17(3):e0011191. doi: 10.1371/journal.pntd.0011191 (PMC10032529; doi:10.1371/journal.pntd.0011191)

Densitometry analysis

Chemiluminescence was recorded with the C-DiGit scanner (LI-COR), and bands were quantified and normalized against the input using ImageJ and ImageLab 6.1 (Bio-Rad) software. The normalization was performed following the "Western Blot Normalization Using Image Lab™ Software" guide. Results are expressed as mean ± SD (n≥3). * p<0.05, ** p <0.005, One-way ANOVA – Dunnett´s multiple comparison test.

HL-1 cells

Gel N° 1

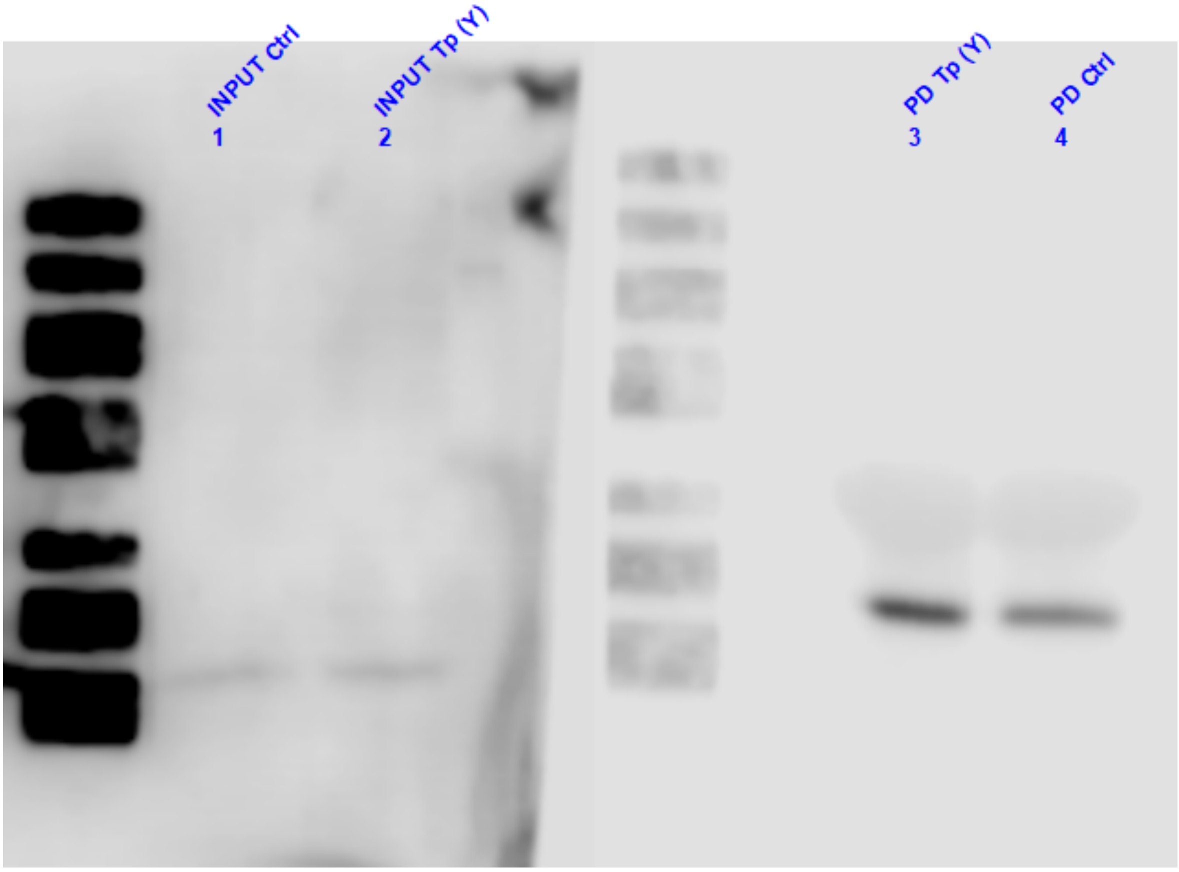

Gel N° 2

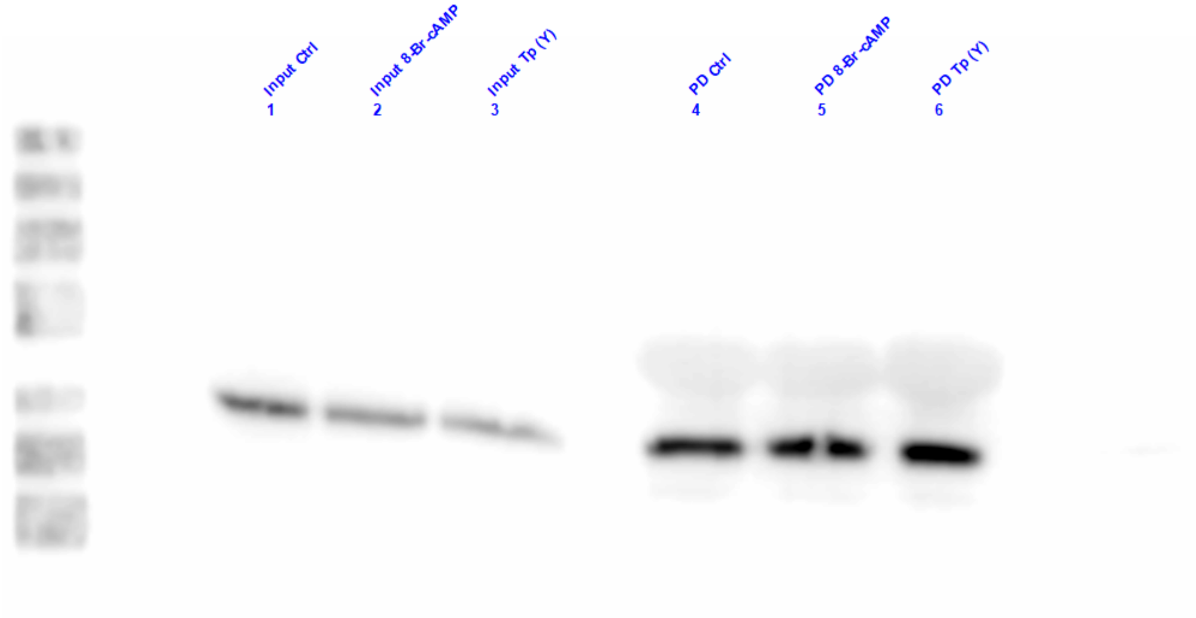
Input Ctrl
1
Input 8-Br-cAMP
2
Input Tp (Y)
3
PD Ctrl
4
PD 8-Br-cAMP
5
PD Tp (Y)
6

Gel N° 3

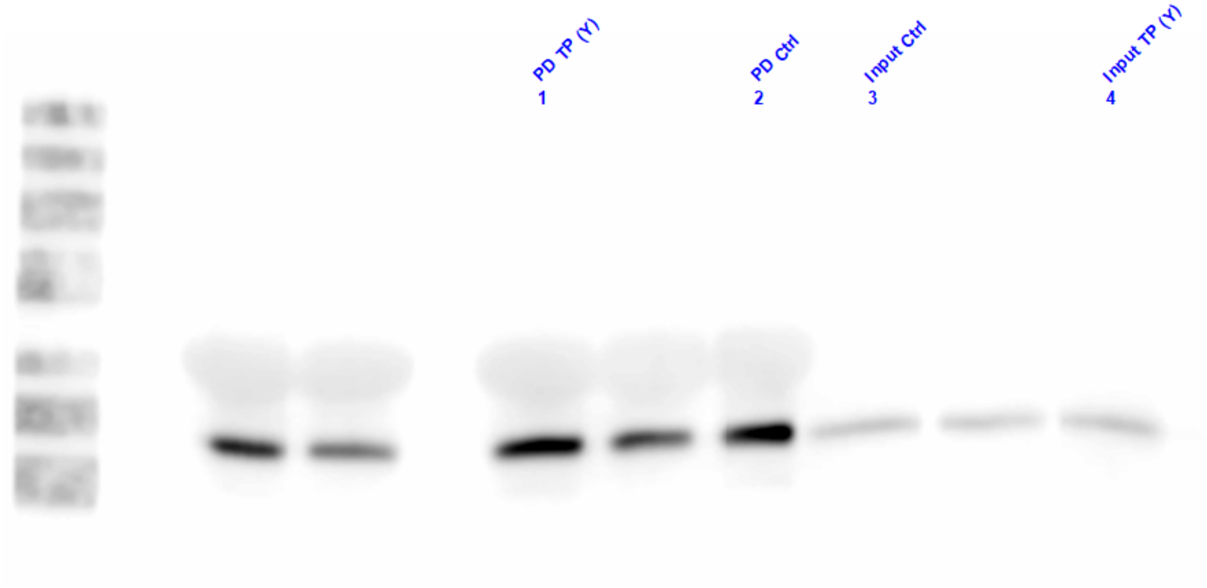
PD TP (Y)
1
PD Ctrl
2
Input Ctrl
3
Input TP (Y)
4

Gel N° 4

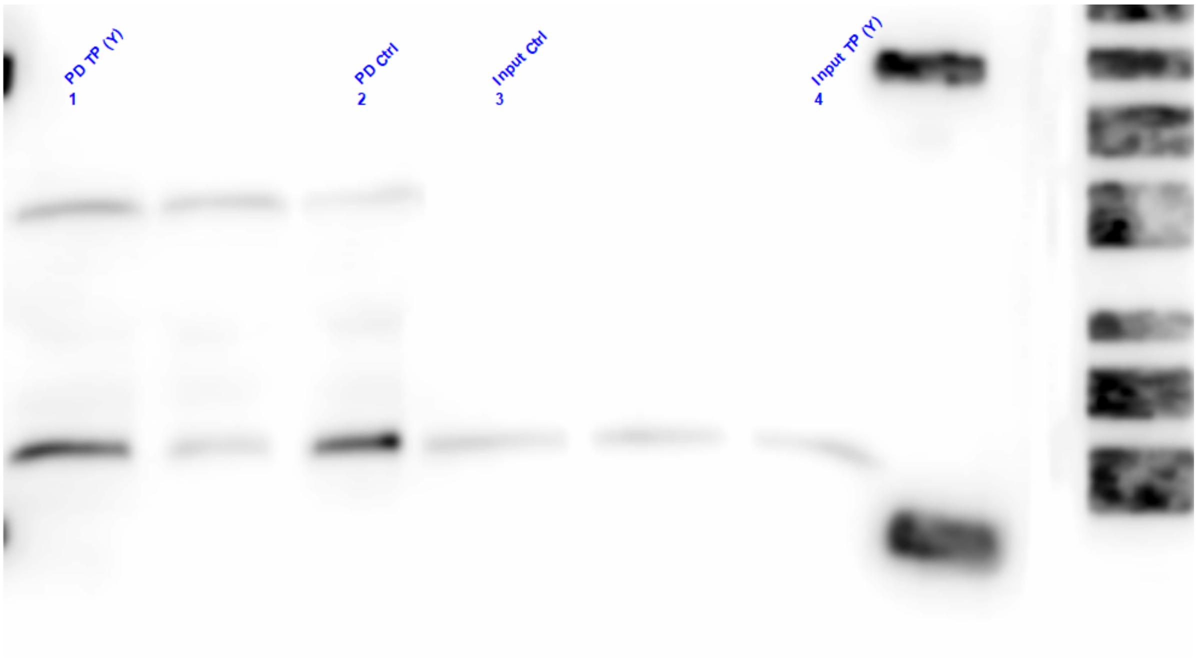
PD TP (Y)
1
PD Ctrl
2
Input Ctrl
3
Input TP (Y)
4

Gel N° 5

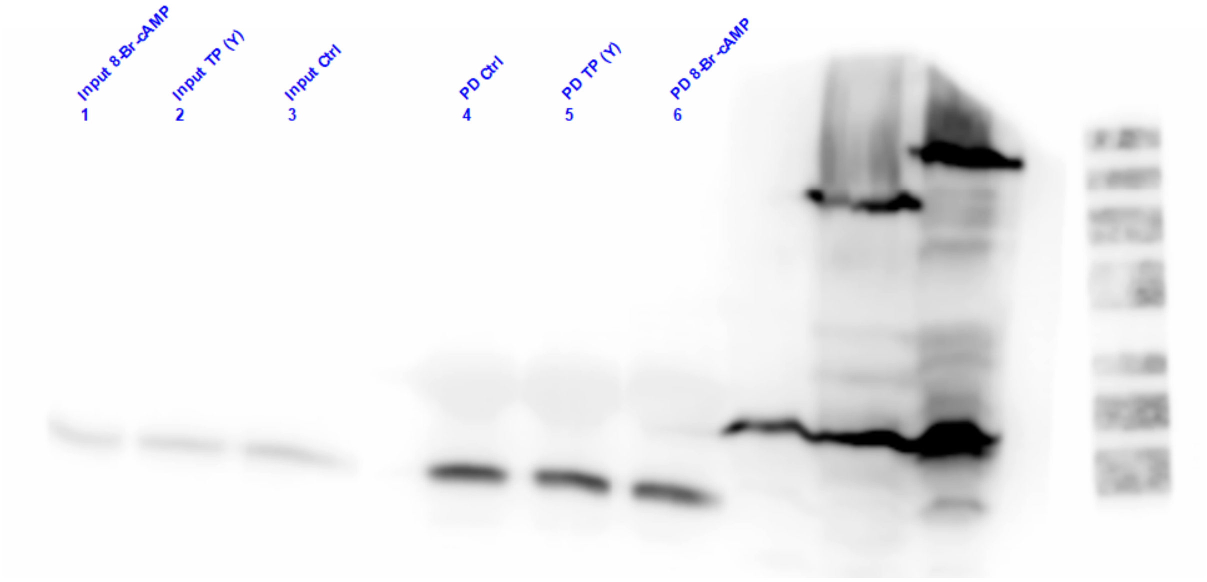
Input 8-Br-cAMP
1
Input TP (Y)
2
Input Ctrl
3
PD Ctrl
4
PD TP (Y)
5
PD 8-Br-cAMP
6

Gel N° 6

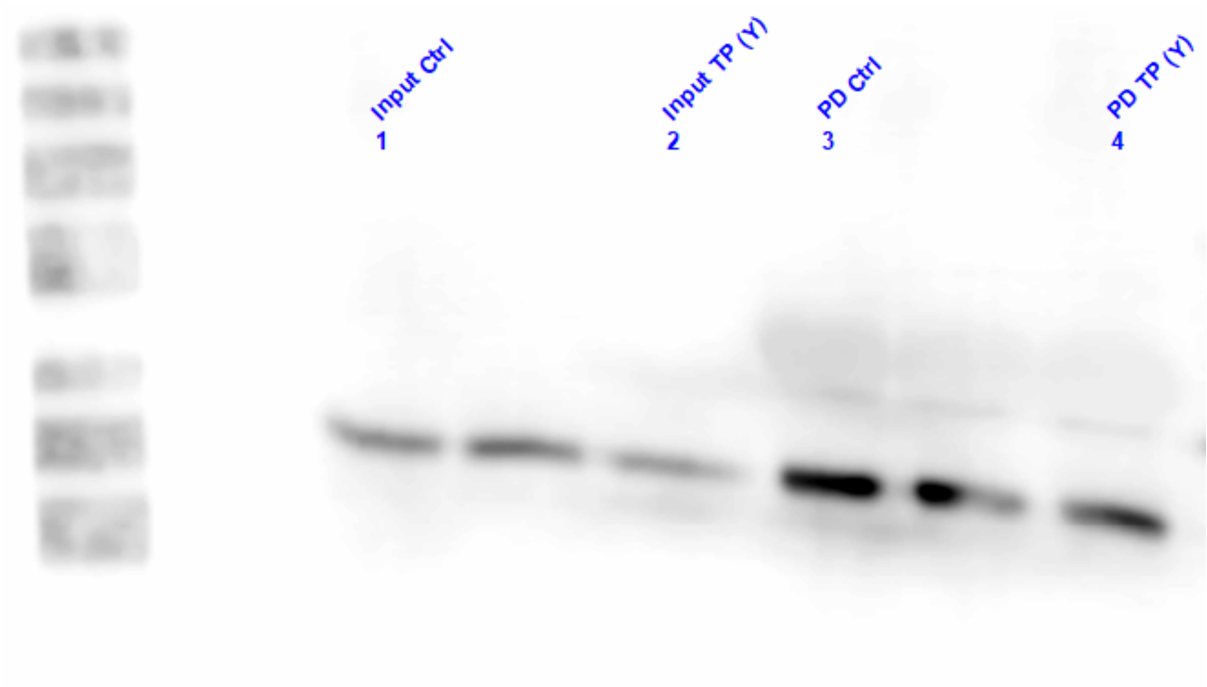

Gel N° 7

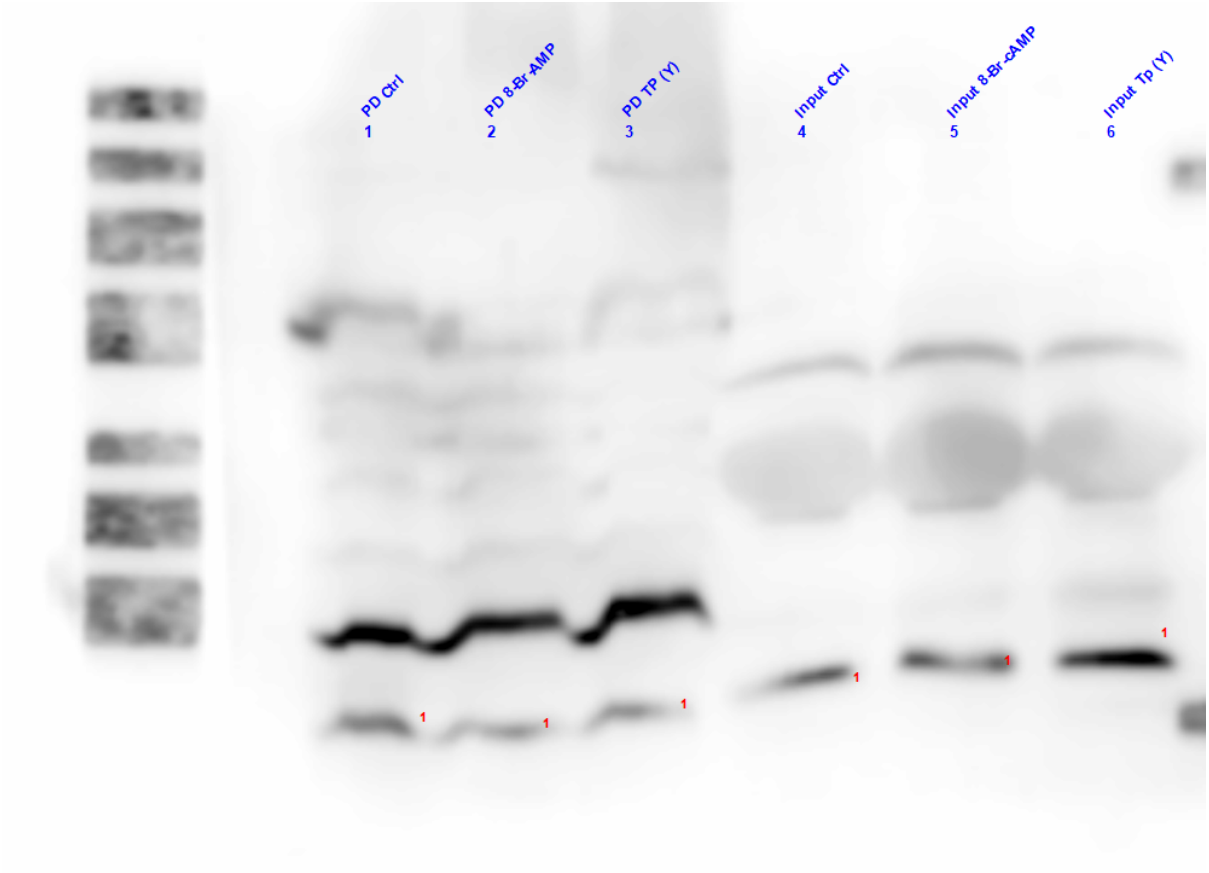

<u>Densitometry</u>

HL-1 (Gel N° 1)											
	Lane	Band No.	Relative Front	**Adj. Volume (Int)**	Volume (Int)	Band %	Lane %	INPUT/INPUT Ctrl	PD/PD Ctrl	**PD/INPUT**	
INPUT Ctrl	1	1	0.760446	**19635**	166551	100	67.46032	1		**Ctrl**	**1**
INPUT Tp (Y)	2	1	0.754875	**19680**	124148	100	54.17607	1.002291826		**Tp (Y)**	**1.3585937**
PD TP (Y)	3	1	0.662953	**93375**	190800	100	78.69785		1.3617074		
PD Ctrl	4	1	0.671309	**68572**	160923	100	72.75775		1		

HL-1 (Gel N° 2)											
	Lane	Band No.	Relative F	**Adj. Volum**	Volume (I	Band %	Lane %	INPUT/INPUT Ctrl	PD/PD Ctrl	**PD/INPUT**	
INPUT Ctrl	1	1	0.600928	**76260**	187860	100	96.81228	1		**Ctrl**	**1**
INPUT 8-Br-cAMP	2	1	0.607889	**55930**	168730	100	99.49833	0.733412012		**8-Br-cAMP**	**1.6609486**
INPUT Tp (Y)	3	1	0.610209	**43845**	160395	100	98.99749	0.574940991		**Tp (Y)**	**2.007365**
PD Ctrl	4	1	0.614849	**115828**	255484	100	94.94721		1		
PD 8-Br-cAMP	5	1	0.600928	**141097**	327947	100	84.87242		1.2181597		
PD TP (Y)	6	1	0.593967	**133679**	277550	100	93.15155		1.1541164		

HL-1 (Gel N° 3)											
	Lane	Band No.	Relative Front	**Adj. Volume (Int)**	Volume (Int)	Band %	Lane %	INPUT/INPUT Ctrl	PD/PD Ctrl	**PD/INPUT**	
PD TP (Y)	1	1	0.744186	**94500**	174600	100	94.52363		1.1161901	**Ctrl**	**1**
PD Ctrl	2	1	0.718023	**84663**	175743	100	96.99605		1	**Tp (Y)**	**1.251542**
INPUT Ctrl	3	1	0.703488	**22950**	98550	100	95.50562	1			
INPUT Tp (Y)	4	1	0.697674	**20468**	103114	100	95.96774	0.891851852			

HL-1 (Gel N° 4)											
	Lane	Band No.	Relative Front	**Adj. Volume (Int)**	Volume (Int)	Band %	Lane %	INPUT/INPUT Ctrl	PD/PD Ctrl	**PD/INPUT**	
PD TP (Y)	1	1	0.715064	**144760**	331980	100	56.94505		1.1668359	**Ctrl**	**1**
PD Ctrl	3	1	0.705989	**124062**	262260	100	83.16708		1	**Tp (Y)**	**1.8433819**
INPUT Ctrl	4	1	0.704174	**37391**	205442	100	98.17518	1			
INPUT Tp (Y)	6	1	0.713249	**23668**	198250	100	19.07571	0.632986548			

HL-1 (Gel N° 5)											
	Lane	Band No.	Relative Front	**Adj. Volume (Int)**	Volume (Int)	Band %	Lane %	INPUT/INPUT Ctrl	PD/PD Ctrl	**PD/INPUT**	
INPUT 8-Br-cAMP	1	1	0.588424	**21024**	93294	100	96.32107	0.546347548		**Ctrl**	**1**
INPUT Tp (Y)	2	1	0.581994	**28756**	113444	100	96.04222	0.747277877		**8-Br-cAMP**	**1.9671624**
INPUT Ctrl	3	1	0.581994	**38481**	140188	100	95.01247	1		**Tp (Y)**	**1.3166222**
PD Ctrl	4	1	0.581994	**123966**	258156	100	81.58879		1		
PD TP (Y)	5	1	0.575563	**121968**	233856	100	80.82061		0.9838827		
PD 8-Br-cAMP	6	1	0.585209	**133233**	277057	100	67.67631		1.0747544		

HL-1 (Gel N° 6)											
	Lane	Band No.	Relative Front	**Adj. Volume (Int)**	Volume (Int)	Band %	Lane %	INPUT/INPUT Ctrl	PD/PD Ctrl	**PD/INPUT**	
INPUT Ctrl	1	1	0.595109	**32544**	85824	100	72.08931	1		**Ctrl**	**1**
INPUT Tp (Y)	2	1	0.570652	**20713**	77539	100	42.56329	0.636461406		**Tp (Y)**	**1.172960012**
PD Ctrl	3	1	0.557065	**86800**	149660	100	59.75904		1		
PD TP (Y)	4	1	0.546196	**64800**	127872	100	61.51743		0.746543779		

HL-1 (Gel N° 7)											
	Lane	Band No.	Relative Front	**Adj. Volume (Int)**	Volume (Int)	Band %	Lane %	INPUT/INPUT Ctrl	PD/PD Ctrl	**PD/INPUT**	
INPUT Ctrl	1	1	0.702381	**61886**	223876	100	23.96694	1		**Ctrl**	**1**
INPUT 8-Br-cAMP	2	1	0.714286	**50046**	200184	100	17.53195	0.808680477		**8-Br-cAMP**	**1.7756627**
INPUT Tp (Y)	3	1	0.67619	**47400**	192000	100	16.73729	0.765924442		**Tp (Y)**	**2.9703365**
PD Ctrl	4	1	0.604762	**67027**	204282	100	77.46637		1		
PD 8-Br-cAMP	5	1	0.57619	**96247**	265742	100	75.6641		1.4359437		
PD TP (Y)	6	1	0.571429	**152490**	345460	100	80.12085		2.2750533		

Global HL-1 analysis:

RAP1-GTP/INPUT								
**HL-1 cells**	**Gel 1**	**Gel 2**	**Gel 3**	**Gel 4**	**Gel 5**	**Gel 6**	**Gel 7**	**average**
**Ctrl**	1	1	1	1	1	1	1	**1**
**8-Br-cAMP**		1.660949			1.967162		1.775663	**1.814056**
**Tp (Y)**	1.358594	2.007365	1.251542	1.843382	1.316622	1.17296	2.970337	**1.491744**

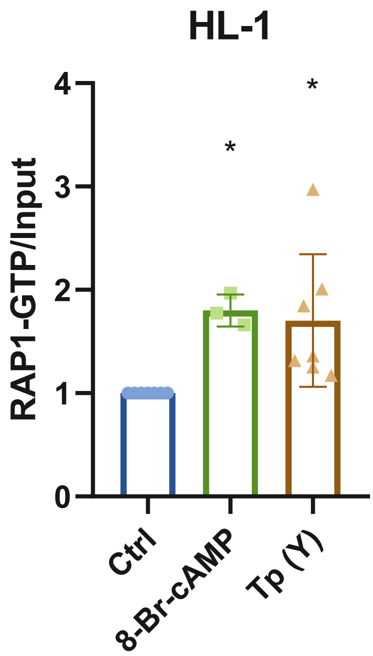

HELA cells

Gel N° 1

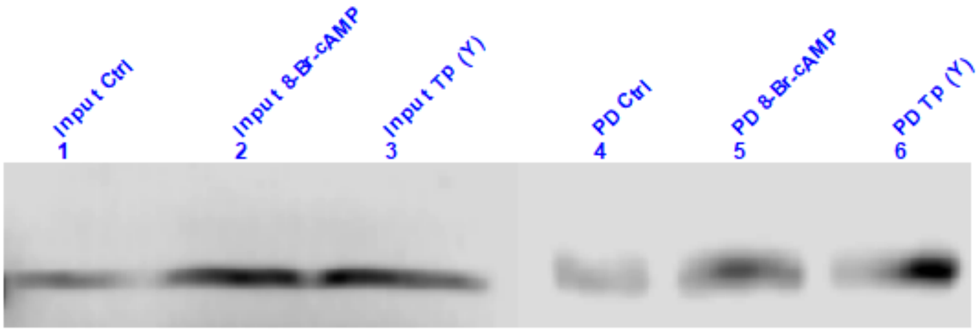

Gel N° 2

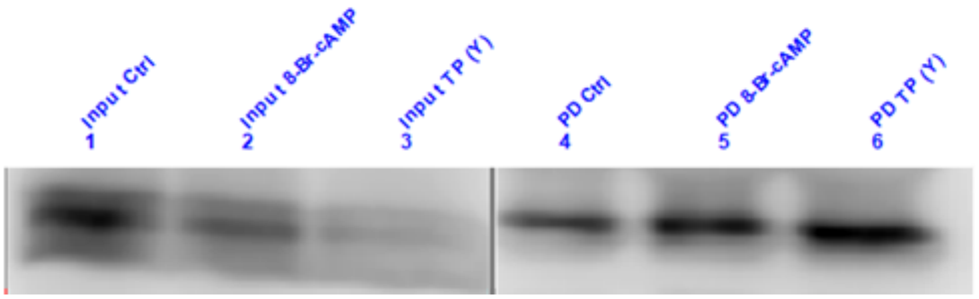

Gel N° 3:

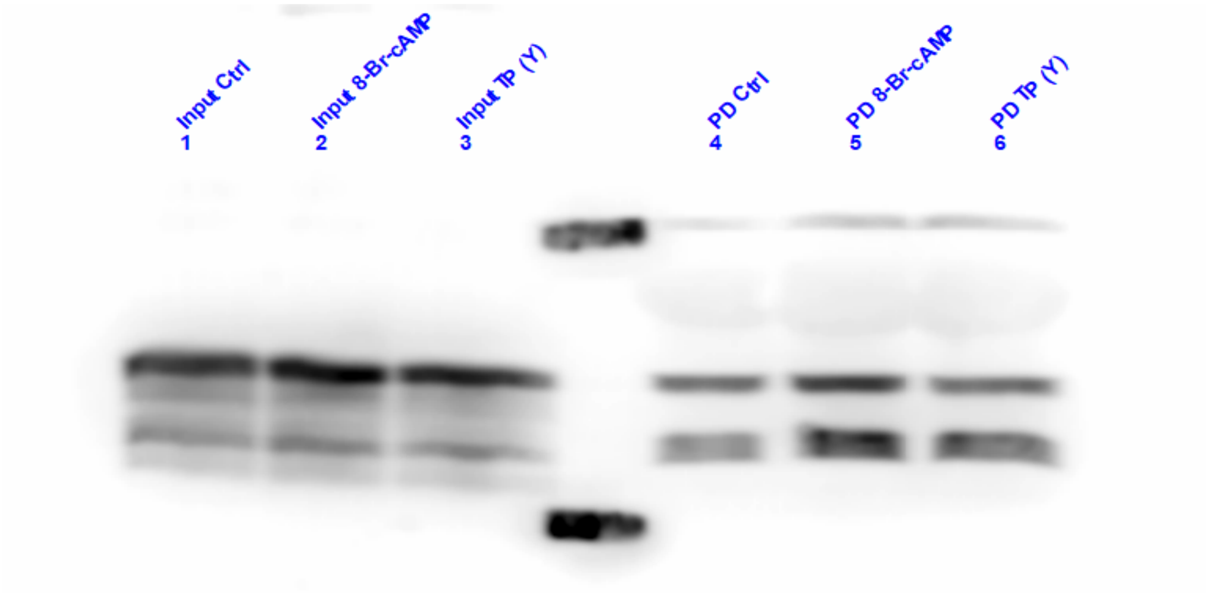

DENSITOMETRY

HELA (Gel N° 1)	Lane	Band No.	Relative Front	Adj. Volume (Int)	Volume (Int)	Band %	Lane %		INPUT/INPUT Ctrl	PD/PD Ctrl	PD/INPUT	
Input Ctrl	1	1	0.627451	10710	29652	100	83.60656		1		**Ctrl**	**1**
Input 8-Br-cAMP	2	1	0.607843	25300	51244	100	75.54945		2.362278245		**8-Br-cAMP**	**1.3403741**
Input TP (Y)	3	1	0.607843	25137	46207	100	85.21595		2.347058824		**Tp (Y)**	**1.5326293**
PD Ctrl	4	1	0.607843	7070	22820	100	99.50739			1		
PD 8-Br-cAMP	5	1	0.568627	22386	45066	100	99.81273			3.1663366		
PD TP (Y)	6	1	0.568627	25432	45144	100	99.82729			3.5971711		

HELA (Gel N° 2)	Lane	Mol. Wt. (KDa)	Relative Front	Adj. Volume (Int)	Volume (Int)	Band %	Lane %		INPUT/INPUT Ctrl	PD/PD Ctrl	PD/INPUT	
Input Ctrl	1	N/A	0.425	66285	66285	100	33.63782		1		**Ctrl**	**1**
Input 8-Br-cAMP	2	N/A	0.525	64998	64998	100	37.70011		0.980583842		**8-Br-cAMP**	**1.4998989**
Input TP (Y)	3	N/A	0.575	51504	51504	100	37.37374		0.777008373		**Tp (Y)**	**1.6205668**
PD Ctrl	4	N/A	0.475	61834	61834	100	50.31491			1		
PD 8-Br-cAMP	5	N/A	0.5	90944	90944	100	51.55556			1.4707766		
PD TP (Y)	6	N/A	0.55	77861	77861	100	42.44124			1.259194		

HELA (Gel N° 3)	Lane	Band No.	Relative Front	Adj. Volume (Int)	Volume (Int)	Band %	Lane %	INPUT/INPUT Ctrl	PD/PD Ctrl	PD/INPUT	
Input Ctrl	1	1	0.694268	13026	46215	100	25.49618	1		**Ctrl**	**1**
Input 8-Br-cAMP	2	1	0.713376	16146	51792	100	20.94082	1.239520958		**8-Br-cAMP**	**1.4283466**
Input TP (Y)	3	1	0.719745	9555	46605	100	16.11842	0.733532934		**Tp (Y)**	**2.0306745**
PD Ctrl	4	1	0.713376	24297	51207	100	42.72977		1		
PD 8-Br-cAMP	5	1	0.700637	43017	76011	100	53.7001		1.7704655		
PD TP (Y)	6	1	0.707006	36192	68835	100	55.60216		1.4895666		

Global HELA analysis:

RAP1-GTP/INPUT				
**HELA**	**Gel 1**	**Gel 2**	**Gel 3**	**average**
**Ctrl**	1	1	1	**1**
**8-Br-cAMP**	1.340374	1.499899	1.428347	**1.422873**
**Tp (Y)**	1.532629	1.620567	2.030674	**1.727957**

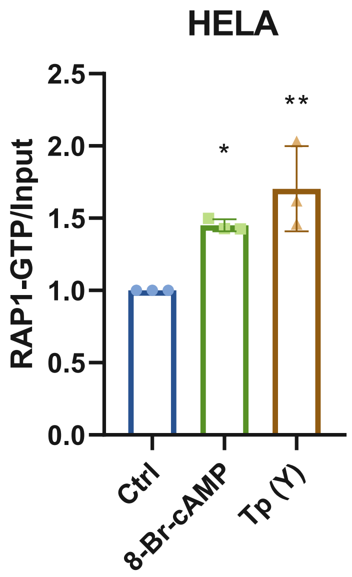

NRK cells

Gel N° 1

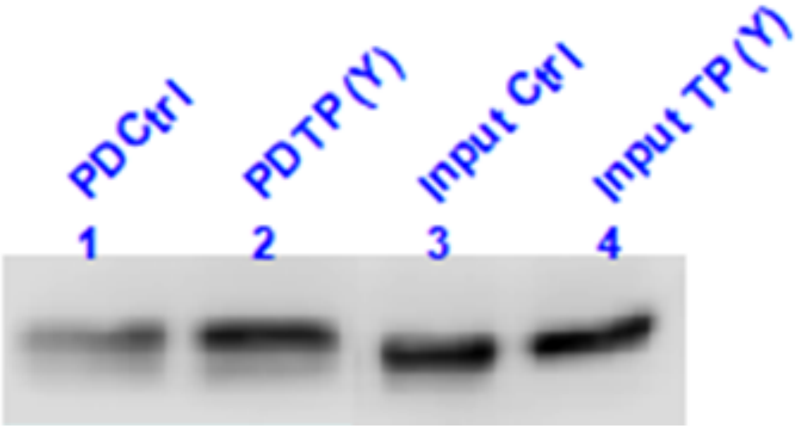

Gel N° 2

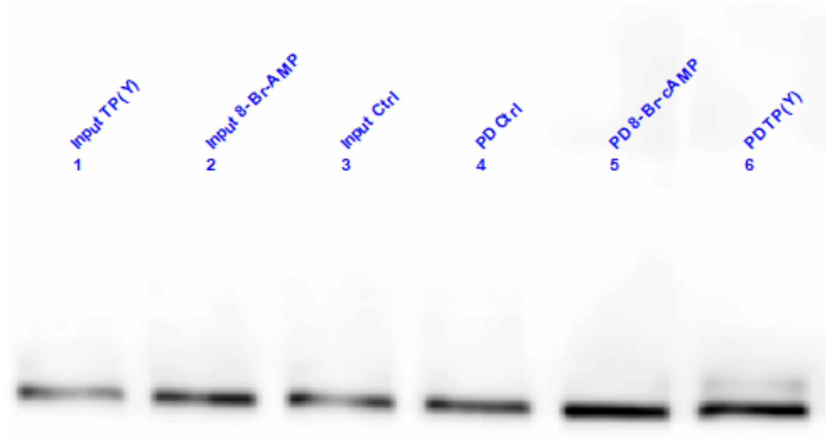

Gel N° 3

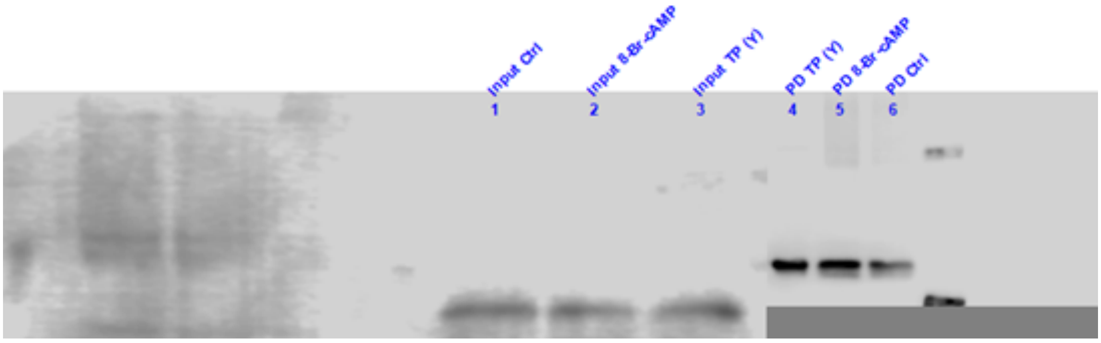

<u>DENSITOMETRY</u>

**NRK (Gel N° 1)**										
	Lane	Relative Front	Adj. Volume (Int)	Volume (Int)	Band %	Lane %	INPUT/INPUT Ctrl	PD/PD Ctrl	**PD/INPUT**	
PD Ctrl	1	0.44186	22099	33538	100	61.67048		1	**Ctrl**	**1**
PD TP (Y)	2	0.418605	52946	65136	100	64.55412		1.9421552	**Tp (Y)**	**2.1584876**
Input Ctrl	3	0.534884	37037	50209	100	77.2973	1			
Input TP (Y)	4	0.44186	33325	47558	100	75.98039	0.8997759			

**NRK (Gel N° 2)**											
	Lane	Relative Front	Adj. Volume (Int)	Volume (Int)	Band %	Lane %		INPUT/INI	PD/PD Ctr	**PD/INPUT**	
Input TP (Y)	1	0.81	16205	30100	100	87.19397		0.760156		**Ctrl**	**1**
Input 8-Br-cAMP	2	0.83	24876	37152	100	83.45411		1.166901		**8-Br-cAMP**	**1.221396**
Input Ctrl	3	0.83	21318	37620	100	88.9065		1		**Tp (Y)**	**1.708871**
PD Ctrl	4	0.85	23598	36670	100	91.45803			1		
PD 8-Br-cAMP	5	0.87	33633	45843	100	74.63054			1.425248		
PD TP (Y)	6	0.86	30654	41457	100	68.4669			1.299008		

**NRK (Gel N° 3)**											
	Lane	Relative Front	Adj. Volume (Int)	Volume (Int)	Band %	Lane %		INPUT/INI	PD/PD Ctr	**PD/INPUT**	
Input Ctrl	1	0.811321	49140	147780	100	75.31035		1		**Ctrl**	**1**
Input 8-Br-cAMP	2	0.811321	38595	114042	100	73.11321		0.785409		**8-Br-cAMP**	**1.83991**
Input TP (Y)	3	0.811321	47802	152613	100	63.06749		0.972772		**Tp (Y)**	**1.439842**
PD TP (Y)	4	0.572327	46137	71838	100	88.5479			1.400638		
PD 8-Br-cAMP	5	0.566038	47601	69823	100	83.22581			1.445082		
PD Ctrl	6	0.572327	32940	62100	100	89.15956			1		

Global NRK analysis:

RAP1-GTP/INPUT				
**NRK**	**Gel 1**	**Gel 2**	**Gel 3**	**average**
**Ctrl**	1	1	1	**1**
**8-Br-cAMP**		1.221396	1.83991	**1.530653**
**Tp (Y)**	2.158488	1.708871	1.439842	**1.769067**

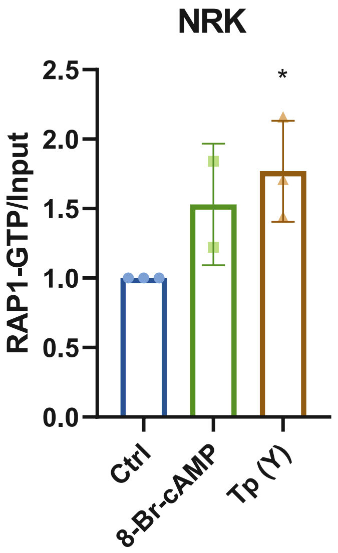

Supplement: S1 Fig — Chemiluminescence was recorded with the C-DiGit scanner (LI-COR), and bands were quantified and normalized against the input using ImageJ and ImageLab 6.1 (Bio-Rad) software. The normalization was performed following the “Western Blot Normalization Using Image Lab Software” guide. Results are expressed as mean ± SD (n≥3). * p<0.05, ** p <0.005, One-way ANOVA–Dunnett´s multiple comparison test. (PDF) [file pntd.0011191.s001.pdf]
