# Supplementary figures and images for: Host Cell Rap1b mediates cAMP-dependent invasion by Trypanosoma cruzi
Source: PLoS Negl Trop Dis. 2023 Mar 10;17(3):e0011191. doi: 10.1371/journal.pntd.0011191 (PMC10032529; doi:10.1371/journal.pntd.0011191)

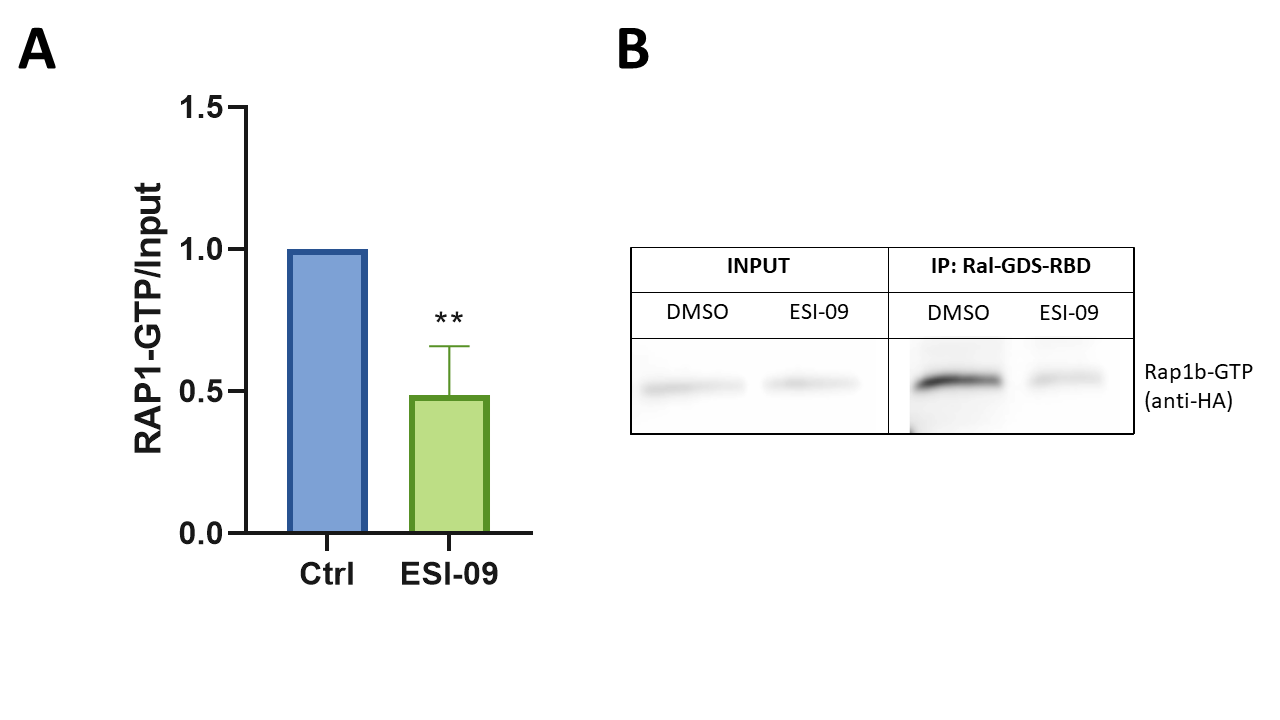

Supplement: S2 Fig — A) HA-Rap1 transfected HELA cells were incubated for 2 h 37.5uM ESI-09 or 0.1% DMSO. Then, cells were lysed and pull-down assay with glutathione-agarose resin performed for 1 h at 4°C. Resin was washed and eluted with cracking buffer for WB analysis. B) Bands were quantified and normalized against the input using ImageJ cell software. Results are expressed as mean ± SD (n≥3). ** p<0.01, t student test. (TIFF) [file pntd.0011191.s002.tiff]

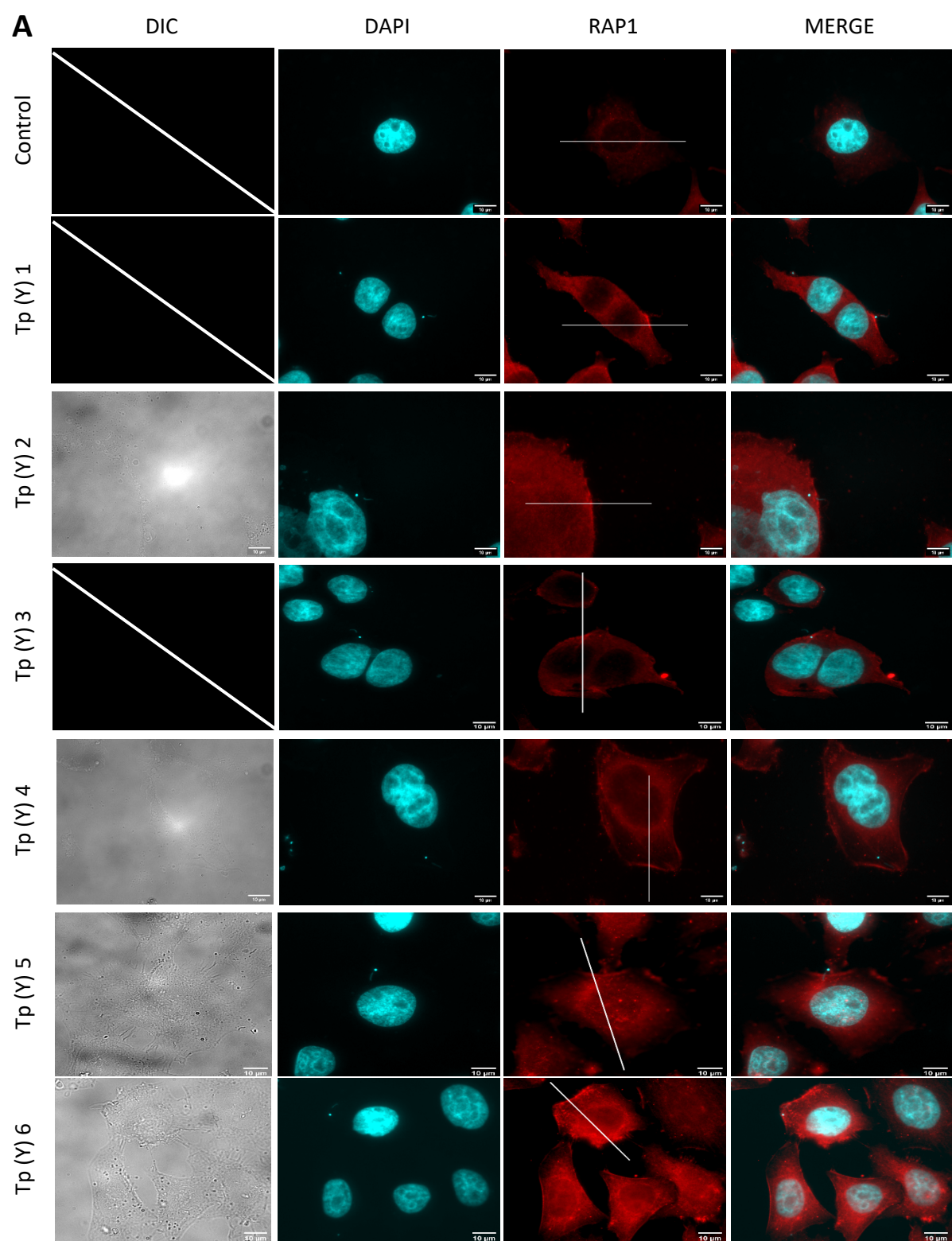

**B. Line profiles**

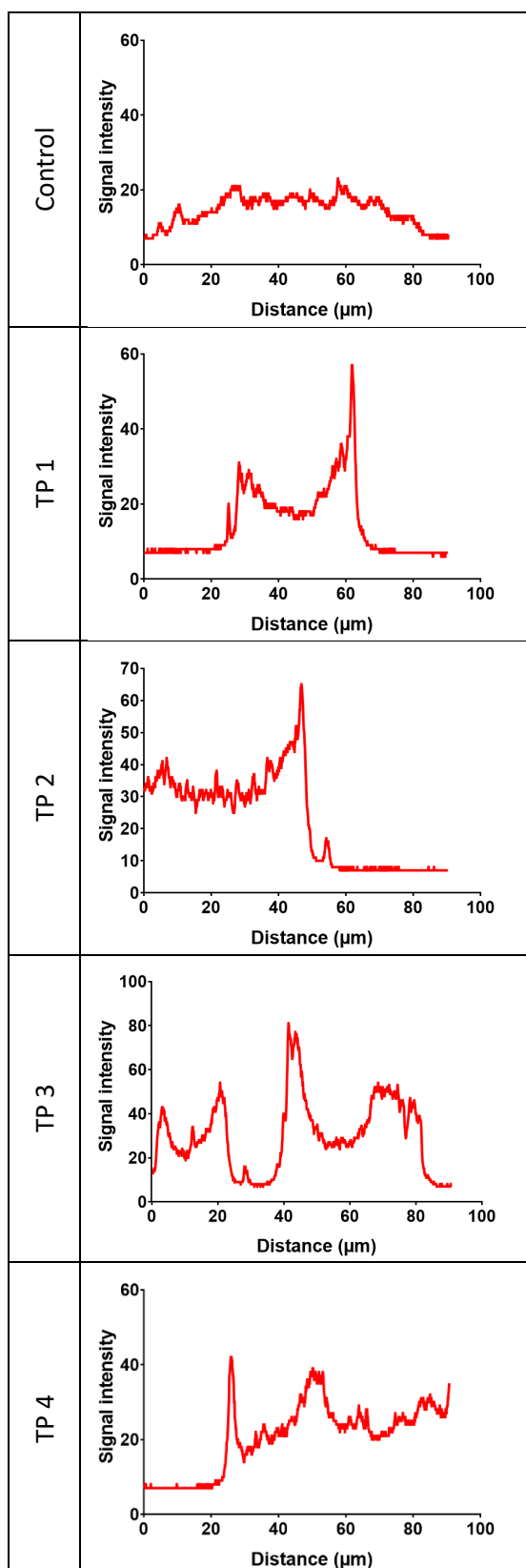

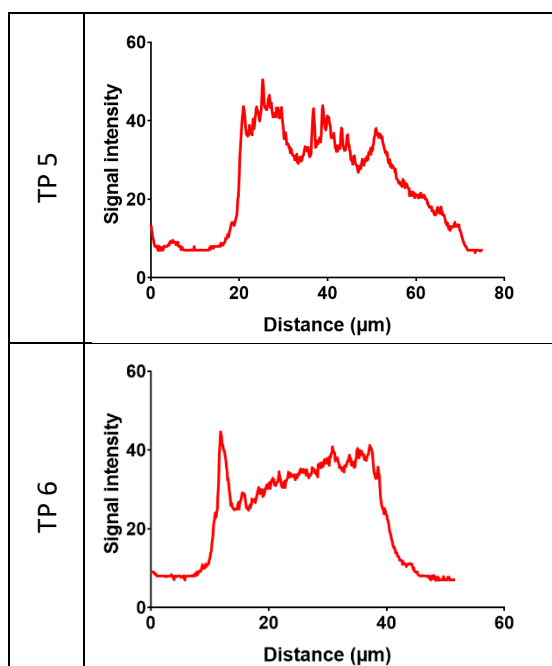

Supplement: S3 Fig — A) Cells were infected for 5 to 15 min with (Tp Y) trypomastigotes from T. cruzi Y strain or mock infected (Control) and then fixed and incubated with primary antibody against Rap1 protein and a secondary antibody conjugated to Alexa594. B) Line profiles obtained from the line shown in the Rap1 quadrant. Photos were taken with a fluorescence microscope. Only a representative image is shown. Scale bar: 10 μm. (PDF) [file pntd.0011191.s003.pdf]

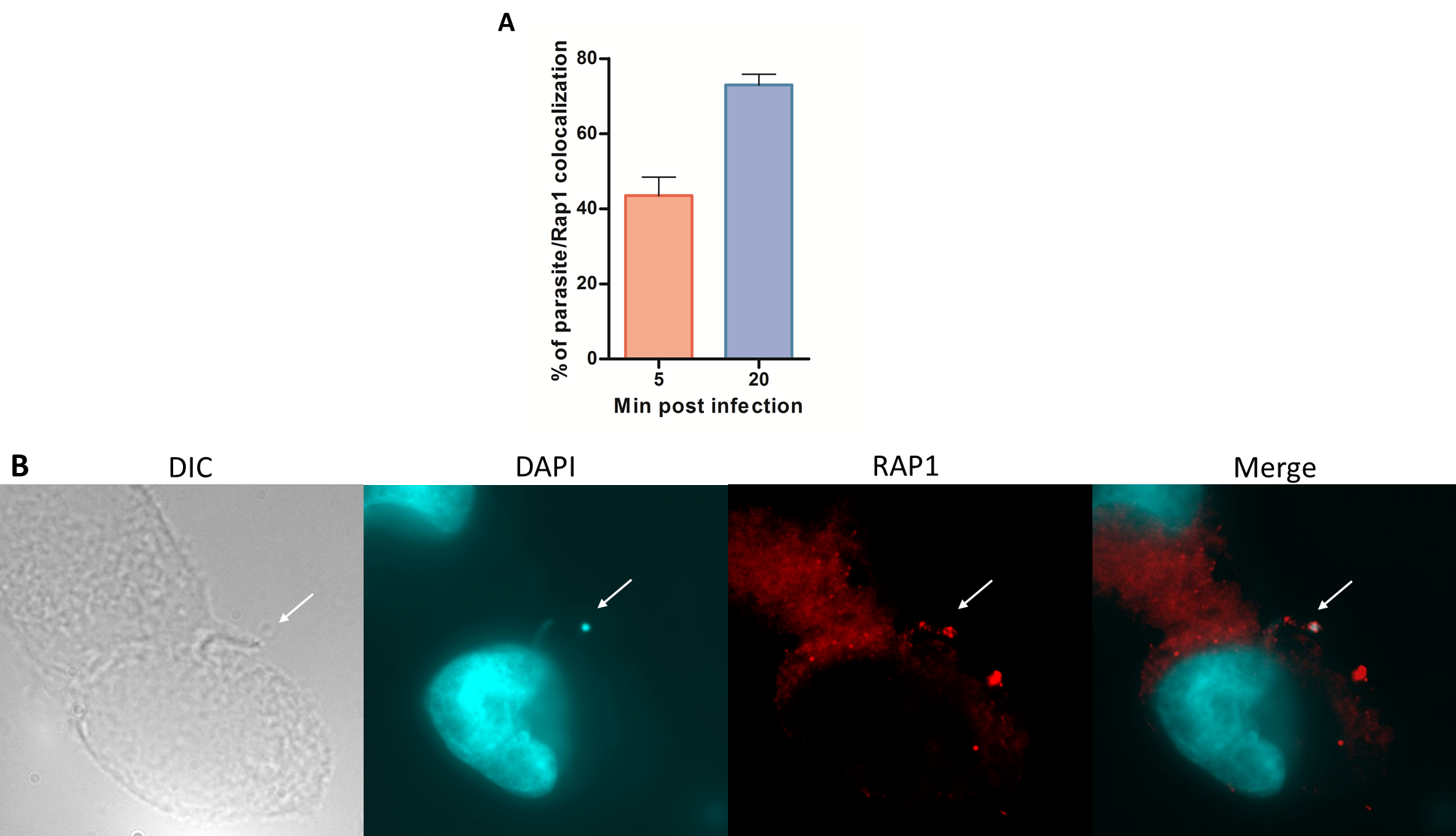

Supplement: S4 Fig — A) HeLa cells were incubated with trypomastigotes and the percentage of infected cells with Rap1 positive signal at the site of parasite entry were quantified at 5 and 20 minutes. Quantification was performed counting at least 30 infected cells of each time point in 3 independent experiments. * p < 0.01 (t test). B) Immunofluorescence of HL-1 cells were infected for 20 min with trypomastigotes from T. cruzi Y strain (Tp Y) (20:1 parasite to cell ratio) or mock infected (Control) and then fixed and incubated with primary antibody against Rap1 protein and a secondary antibody conjugated to Alexa594. Photos were taken with a fluorescence microscope. (TIF) [file pntd.0011191.s004.tif]

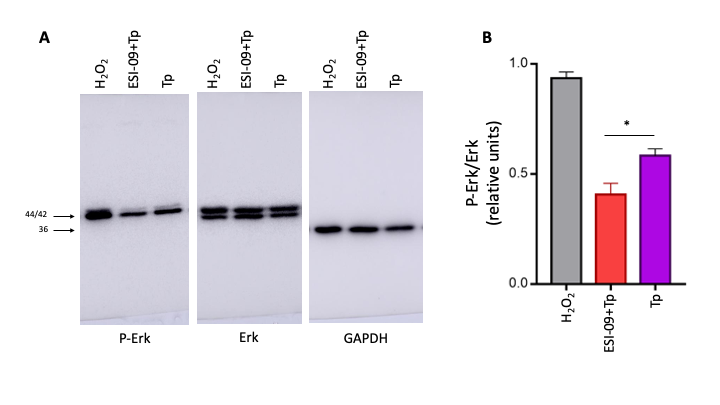

Supplement: S6 Fig — A) HeLa cells were pretreated with H2O2 (750 mM, 5 min) or ESI-09 (37.5mM, 30 min), then cells were washed and incubated with trypomastigotes for 2 hours. Cells were washed and lyzed. WB with ERK, P-ERK or GAPDH antibodies were performed. B) Erk and P-Erk band density were normalized to GAPDH, then P-Erk expression was relativized to total Erk. Band density were quantified using ImageJ. * p < 0.01 (t test). (TIFF) [file pntd.0011191.s006.tiff]
