# Supplementary material for: Host Cell Rap1b mediates cAMP-dependent invasion by Trypanosoma cruzi
Source: PLoS Negl Trop Dis. 2023 Mar 10;17(3):e0011191. doi: 10.1371/journal.pntd.0011191 (PMC10032529; doi:10.1371/journal.pntd.0011191)

ERK phosphorylation.

NRK cells were incubated for 2 h with trypomastigotes from *T. cruzi* Y strain (Tp Y), treated with 750 µM H_2O_2 for 5 min (positive control) or mock infected (Control). Then, cells were lysed and cracking buffer added for WB analysis.

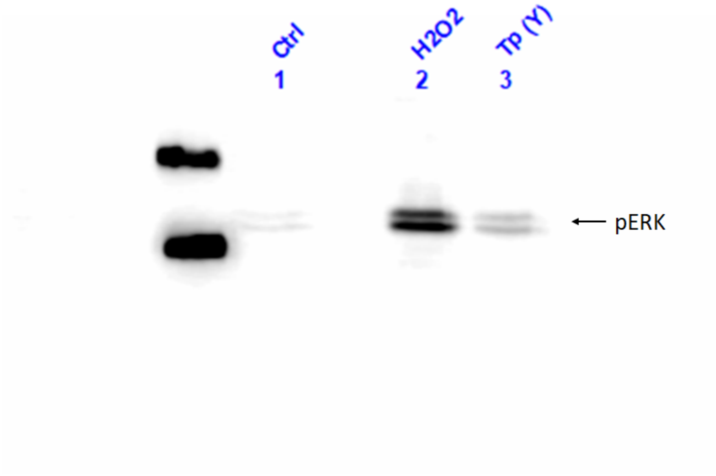

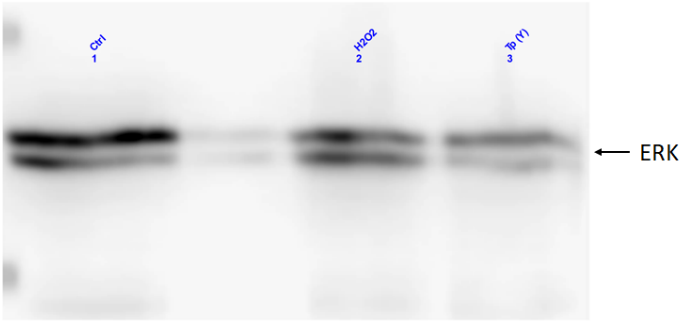

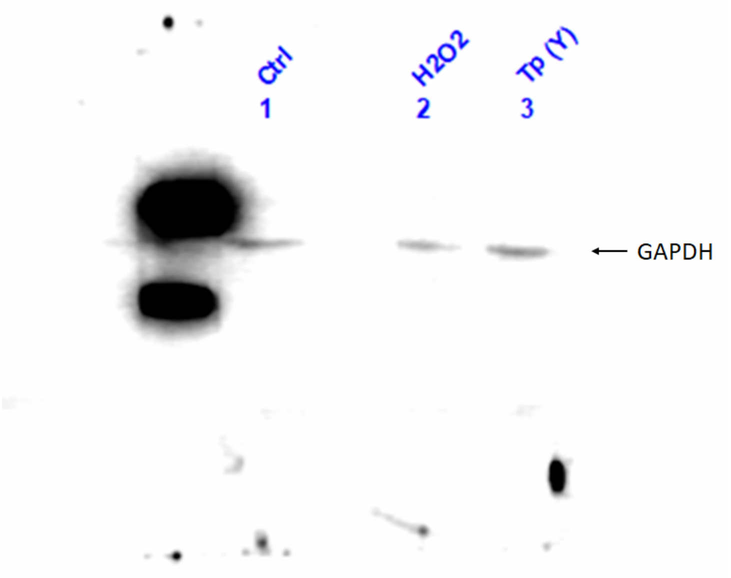

HL-1 cells were incubated for 2 h with trypomastigotes from *T. cruzi* Y strain (Tp Y) or mock infected (Control). Then, cells were lysed and cracking buffer added for WB analysis.

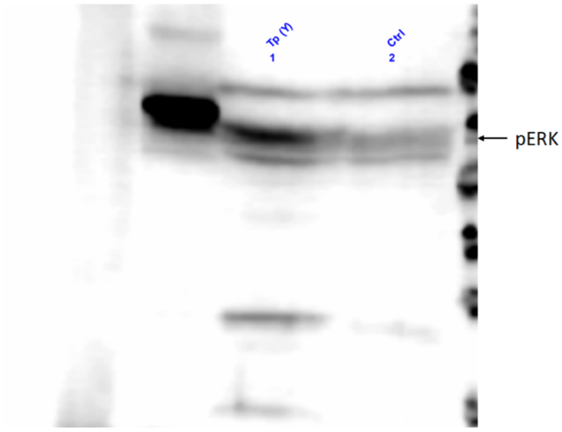

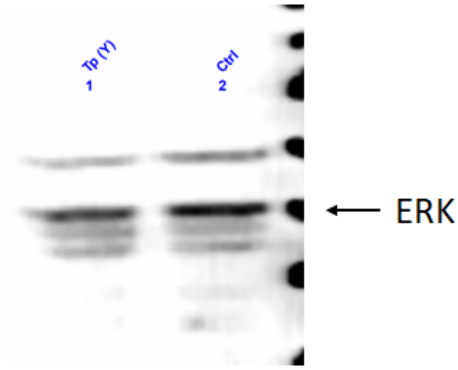

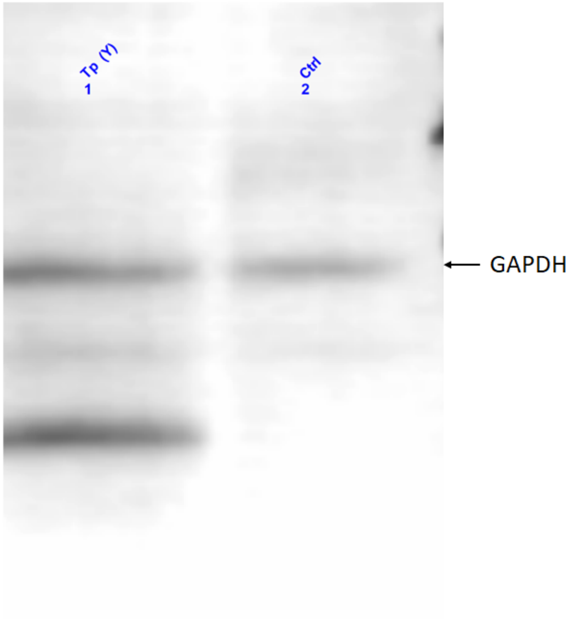

Supplement: S5 Fig — Upper) NRK cells were incubated for 2 h with trypomastigotes from T. cruzi Y strain (Tp Y), treated with 750 μM H2O2 for 5 min (positive control) or mock infected (Control). Then, cells were lysed and cracking buffer added for WB analysis. Lower) HL-1 cells were incubated for 2 h with trypomastigotes from T. cruzi Y strain (Tp Y) or mock infected (Control). Then, cells were lysed and cracking buffer added for WB analysis. (PDF) [file pntd.0011191.s005.pdf]
