# Supplementary material for: Host Cell Rap1b mediates cAMP-dependent invasion by Trypanosoma cruzi
Source: PLoS Negl Trop Dis. 2023 Mar 10;17(3):e0011191. doi: 10.1371/journal.pntd.0011191 (PMC10032529; doi:10.1371/journal.pntd.0011191)
Supplement: Attachment — Submitted filename: Response to reviewers.docx [file pntd.0011191.s007.docx]

Author responses to reviewer’s comments

In first place, authors would like to thank reviewers for the detailed analysis of the manuscript and all suggestions.

Reviewer #1: The methods are satisfactory and include commercial information on reagents used. Nonetheless, a few points need to be reviewed, for example:
1) Although the figures’ subtitles describe the sample size, it is important to describe them in the methods as well. It is important to express if the experiments are independent and if each experiment was done in triplicate.

2) Some of the information needs to be reported in the methods, for instance, the g force, and how much time was used in centrifugation for obtaining the trypomastigotes (line 84). In line 86, it is important to describe the size of the glass cover slides.
3) In section “GST Pull-down” (line 111), the relationship between glutathione and the question is not clear. It is crucial to explain how this methodology works and the outcome’s relation to the question.
4) The Resazurin sodium salt dye is commonly used as a fluorogenic oxidation-reduction indicator in a variety of cells. Therefore, it is necessary to describe how this reagent was used in your experiments to quantify the trypomastigotes released in the supernatant of the experiments.
Suggestions were considered and text modified accordingly.

Reviewer #2: (No Response)

Reviewer #3: The main goal of the manuscript is to describe the pathway by which cAMP/Epac participate in T.cruzi TCTs mechanism of host cell entry. Based on previous results from the group and the literature, the main hypothesis is that the pathway involves the activation of Rap1b, an effector downstream of Epac pathway. So the objectives are clear and most of the experimental design is suited to test this main hypothesis. There is, though, one experiment that was not suited to show the point that authors wanted to test. They have used a step at the end of parasite intracellular life cycle (trypomastigote release from infected cells) to indirectly evaluate the role of rap1b in parasite invasion, which is not suited to show this point, since there are direct methods, used in the present manuscript , which would incontestably prove this point. Some of the statistical analyses (figure2, for example) could be reviewed, since there are differences that should be statistically significant and do not show significancy with the test used. All the other points, such as sample size and methods description are ok.

We completely agree with the reviewer in this point. At the time we were looking for a high throughput reliable technique in comparison to cell counting in the microscope. It is more than clear that it was a mistake to change the method, since, as the reviewer, we “have used a step at the end of parasite intracellular life cycle (trypomastigote release from infected cells) to indirectly evaluate the role of rap1b in parasite invasion”. However, the results shown here, PKA-mediated phosphorylation of Rap1b induced a decrease of invasion, were consistent with findings previously reported (Musikant D, et al. Host Epac1 is required for cAMP-mediated invasion by Trypanosoma cruzi. Molecular and Biochemical Parasitology. 2017;211:67–70) and preliminary data obtained counting infected cells. Unfortunately, at the present we have no funding whatsoever to performed the requested assays. If necessary, this figure could be removed from the manuscript.

--------------------

**Results**


-Does the analysis presented match the analysis plan?

-Are the results clearly and completely presented?

-Are the figures (Tables, Images) of sufficient quality for clarity?


Reviewer #1: The results were well-presented and were described adequately. The figures’ subtitles were also clear, but in the caption for figure 4, the ratio of the parasite to cell was missing. Moreover, the NRK graphs in figure 1 require 8-Br-AMPc to be changed to 8-Br-cAMP.

All suggestions were considered and text changed in accordingly.

Reviewer #2: (No Response)

Reviewer #3: All the experiments were designed to show the points they want to prove and, as mentioned, are adequate to make their point, except for figure 5. The data is well presented, even though there are some important points of discussion, which will be addressed in the general comments. The figures are well presented and of sufficient quality for clarity.

--------------------

**Conclusions**


-Are the conclusions supported by the data presented?

-Are the limitations of analysis clearly described?

-Do the authors discuss how these data can be helpful to advance our understanding of the topic under study?

-Is public health relevance addressed?


Reviewer #1: The conclusions are favorable and are supported by the data presented in the article. Although the experimental design using cell lines is adequate, the manuscript would benefit from discussing the limitations of the use of cell lineage to answer the questions presented.

A breakdown of the limitations of the use of cell lineage has been added at the beginning of the discussion.

Reviewer #2: (No Response)

Reviewer #3: Part of the conclusions are supported by the data presented. There are some relevant points, though, that need to be addressed before a final conclusion is made. The discussion of the data is relevant and address the important points of the study for the field.

--------------------

**Editorial and Data Presentation Modifications?**


Use this section for editorial suggestions as well as relatively minor modifications of existing data that would enhance clarity. If the only modifications needed are minor and/or editorial, you may wish to recommend “Minor Revision” or “Accept”.

Reviewer #1: The article is useful to understand more about the mechanism of T. cruzi invasion, a fundamental aspect of Chagas disease, which is still not completely clear. Moreover, a vaccine for this disease has still not been developed, and the treatment is extremely toxic for the patients. Thus, to know more about the possibilities of intervention in the relationship between T. cruzi and cells becomes an interesting point of investigation.
The main point that needs to be reviewed is the introduction.
The introduction of the article “Host Cell Rap1b mediates cAMP-dependent invasion by Trypanosoma cruzi” needs to be rewritten. The objectives and hypothesis are not clear. Furthermore, it is necessary to put in the introduction more information about the cAMP/Epac/Rap1 pathway, especially about Rap1b, which is one of the study’s questions. The information presented in lines 194-217 is important to understand the article, and it would be enlightening if presented in the introduction with this clarity and organization.

It is not quite clear for the authors the modifications suggested by this reviewer for the introduction since “information presented in lines 194-217” is already in the introduction (lines 28 to 67).

The objective of the work (lines 74-76) “…investigated the involvement of two known effectors, Rap1b and ERK, as potential mediators in the cAMP/Epac-dependent invasion by T. cruzi and the role of PKA-dependent Rap1b phosphorylation.” could be modified if necessary.

Reviewer #2: (No Response)

Reviewer #3: no comments

--------------------

**Summary and General Comments**


Use this section to provide overall comments, discuss strengths/weaknesses of the study, novelty, significance, general execution and scholarship. You may also include additional comments for the author, including concerns about dual publication, research ethics, or publication ethics. If requesting major revision, please articulate the new experiments that are needed.

Reviewer #1: The study entitled “Host Cell Rap1b mediates cAMP-dependent invasion by Trypanosoma cruzi” is essentially about the importance and participation of some molecules in Trypanosoma cruzi host cell invasion. Using immunofluorescence and western blot, the authors demonstrate the involvement of the MEK/ERK signaling downstream of cAMP/Epac/Rap1b-mediated invasion in different cell line hosts. Understanding this invasion mechanism and possible interventions is of great value for Chagas disease. With some adjustments, **the introduction will be more directed to the study proposed by the authors, and even more understandable for the reader,** as already mentioned. The information on some points of the methodology is important so that other researchers can reproduce the experiments and so that readers can understand the techniques used. The results obtained corroborate the study's hypothesis and questions (even if they are not completely clear), and the conclusions allow the reader to understand the importance of this study, although it was conducted in cell lines and this fact has not been discussed.
Introduction and discussion have been modified.

Reviewer #2: Ferri and Edreira sought to evaluate the participation of Rap1b and its GEF, Epac (cAMP-activated), on host cell invasion by tissue-cultured derived trypomastigotes of Trypanosoma cruzi.
Authors provide however insufficient results to fully support this hypothesis.
The manuscript demands substantial improvement on quality and on experimental evidences to be considered for publication and to make an impact in the community.

In agreement with reviewers 1 and 3, we believe our results are strong, although figure 5 and related discussion and conclusions, might have to be removed from the manuscript.

Major concerns below:
Line 145: “high levels of cAMP induced by..” no experiment showing increased levels of cAMP was presented. Authors could show the dose-dependent response in host cell cAMP levels upon treatment with these drugs and correlate it with parasite internalization.

Elevation in cytosolic cAMP levels induced by trypomastigotes of *T. cruzi* has been previously shown (Rodriguez A, et at, cAMP regulates Ca2+-dependent exocytosis of lysosomes and lysosome-mediated cell invasion by trypanosomes. J Biol Chem. 1999;274(24):16754-9 and Musikant D, et al. Host Epac1 is required for cAMP-mediated invasion by Trypanosoma cruzi. Molecular and Biochemical Parasitology. 2017;211:67–70). Results shown below corroborates this increase in HL-1.

In addition, Epac and Rap1b activation shown in this manuscript, are in accordance with the intracellular elevation of cAMP, since Epac is a well described cAMP effector and Rap1b a downstream effector of the pathway.

HL-1 cells were pretreated with the Phosphodiesterase Inhibitor IBMX (3-Isobutyl-1-methylxanthine) (Sigma) (1.0 mM, 5 minutes). Cells were washed and incubated with or without (NI) tripomastigotes (Y strain moi 100:1, 30 minutes). After that cells were lzed and processed for intracellular cAMP quantification as indicated by the kit manufacturer (cAMP-Glo Assay, Promega).

Line 149: Participation of Epac on T. cruzi invasion should be further corroborated by another approach such as Epac protein knockdown or knockout techniques.

Epac participation has been demonstrated by using the specific inhibitor ESI-09. In addition, we have previously shown that a permeable version of 15-mer sequence (stearate-KPRACSYDLLLEHQRP-amide peptide) corresponding to the minimal Epac1 ERM binding domain was able to partially block invasion, an observation that is consistent with the involvement in *T. cruzi* invasion of a pool of Epac1 compartmentalized with ERM-actin.

Line 155: Epac inhibitor, ESI-09, should also be included in these studies to address eventual reduced activation of Rap1b.

This result has been added as supplementary Fig1.

Line 157: Different experiments can be explored in different paragraphs/topics. ???

A full stop has been added and different topic are now in a different paragraph.

Line 160: it was not clear why and how the trypomastigote release experiment was done. Were authors aiming to address Rap1b also in parasite intracellular life cycle (multiplication and egress)?

As mention before, at the time we were looking for a high throughput reliable technique in comparison to cell counting in the microscope. It is more than clear that it was a mistake to change the method, since, as the reviewer, we “have used a step at the end of parasite intracellular life cycle (trypomastigote release from infected cells) to indirectly evaluate the role of rap1b in parasite invasion”. However, the results shown here, PKA-mediated phosphorylation of Rap1b induced a decrease of invasion, were consistent with findings previously reported (Musikant D, et al. Host Epac1 is required for cAMP-mediated invasion by Trypanosoma cruzi. Molecular and Biochemical Parasitology. 2017;211:67–70) and preliminary data obtained counting infected cells. Unfortunately, at the present we have no funding whatsoever to performed the requested assays. If necessary, this last figure could be removed from the manuscript.

Line 161: It is not clear what the G12V stands for.

G12V is a single point mutation with a glycine-to-valine substitution at codon 12 of Rap1b. This mutation has been previously described for Rap1b and Ras. An aclaration was added in line 175.

Line 162: “…played a role in the early steps of the establishment of infection…” Can the authors elaborate on that? Do authors suggest that Rap1b, besides invasion, also modulate host Rap1b pathway once parasites are internalized?

A reference was added in which we hypothesized: “It has been shown that an intact, stiff and rapidly remodeling cytoskeleton would be required for the initial steps of parasite invasion and parasite retention [**Host cell actin polymerization is required forcellular retention of Trypanosoma cruzi and early association withendosomal/lysosomal compartments Cell. Microbiol. 6 (9) (2004) 829–838**]. Upon invasion, a time-dependent softening and disassembly of the cytoskeleton dictates the ability of internalized trypomastigotes to remain inside the cell, with changes occurring through PKA-mediated inhibition of the Rho/Rho kinase-signaling pathway [Modulation ofhost cell mechanics by Trypanosoma cruzi, J. Cell. Physiol. 218 (2) (2009)315–322]. The fact that Epac/Rap signaling could participate in reorganization of the cytoskeleton thru activation of RhoA [**Cyclic AMP-Rap1A signaling activates RhoA to inducealpha(2c)-adrenoceptor translocation to the cell surface of microvascularsmooth muscle cells, Am. J. Physiol. Cell Physiol. 303 (5) (2012) C499–511**], allowed us to raise the hypothesis thatcAMP/Epac signaling pathway could be activated by T. cruzi at the initial steps of invasion, promoting Ca2+-dependent exocytosis of lysosomes and cytoskeletal reorganization, triggering lysosome-mediated cell invasion. Once the parasite gained access to the cell, PKA-mediated inhibition of the Epac pathway would abolish F-actin formation and lysosome-mediated invasion. In line with this hypothesis, our results showed that inhibition of PKA increased the percentage of invasion, presumably by deregulation of Epac/Rap1b activity, confirming the requirement of a temporal loses of function of Epac for the establishment of the infection.”

Line 165: Quantification of Rap1b recruitment to the parasite’s invasion sites is needed. Also to be included brightfield channels to spatially correlate parasite-host cell contact zones with Rap1b recruitment. Also, how are these Rap1b recruitments correlated to lysosomes and actin, major drivers of parasite internalization?

Rap1 accumulation and quantification of the recruitment has been added as supplementary figures 2 and 3.

We have not explored the correlation between Rap1b recruitment and lysosome and actin, although it is an interesting relation we would analyse in the future.

Line 167: Authors comment that Rap1b needs to be activated to be recruited by the parasites. To further corroborate this hypothesis authors should include activators and inhibitors of the pathway as in Fig1 (8-Br-cAMP and ESI-09) and do proper quantifications of Rap1 recruitment.

The activation and relocalization of Rap1b proposed is in line with the previous results shown in the manuscript. While is true that using an activator and an inhibitor would have given a more direct result of our hypothesis, we were unable to performed the experiments. Authors think that the present data is strong enough to arrive to the conclusion presented.

Line 174: “To evaluate the role of Rap1b phosphorylation…” in parasite infection? If so, assays as in Fig1 should be performed instead of the trypomastigote release showed.

This sentence refers to host’s Rap1b.

Line 186: ERK activation should be assessed in time-dependent manner upon T. cruzi-host cell interaction because the activation profile can differ between infective (trypomastigote and amastigote) and non-infective (epimastigote) forms (Bonfim-Melo et al., 2015, Ferreira et al., 2016).

While is true that ERK activation time can differ from trypomastigotes to amastigotes and epimastigotes and a distinct profile could be found for each, it is beyond the subject of study of this paper.

Line 193: I agree no additive effects on double inhibition of Epac and ERK further suggests they act on the same pathway. However further experiments could be performed to further corroborate that Epac acts upstream ERK during parasite internalization. For example: does ESI-09 inhibit the parasite-induced ERK activation?
The suggested assay has been added as supplementary figure 4

Fig1 (and all others): It is recommended to use data presentation with individual observations rather than bar graphs. It helps readers to understand for example why it was observed statistical differences in cases of small differences in group means (like in Fig1 NRK) while no statistical difference in cases of big differences in group means (like in Fig2 NRK and HeLa 8Br-cAMP).

Supplementary data has been added with gel quantification and new data.

Fig2: Most WB images are of very poor quality (Fig2 “HL-1”) and with cropped bands. It is highly recommended to present uncropped versions of these data.

Uncropped versions of the WBs have been added

Fig2A: The input panel from HeLa cells shows no consistent Rap1b between the groups. How many experiments are these representative images from? Authors should also provide densitometry analyses of these bands as well as the original images for the cropped and uncropped blots.

Uncropped versions and densitometry of the WBs have been added

Figure3: It is not clear what i, ii and iii stand for. I recommend using A to E labels for all plots.

Labels were changed.

Fig4: It should be shown a couple of parasite-host cell interactions with an inset detailing the most representative one. Also, a scale bar should be included.

Scale bar and supplementary photograph added.

Fig5: figure title states “…effect on invasion.” But apparently the experiment quantifies supernatant release amounts of trypomastigotes after 6 days of infection; this is not directly measuring parasite internalization rather it assesses construct effects on parasite intracellular life cycle/multiplication.

This has been discussed above

Fig5: It is not clear which group was used as control for the statistics.

Control was cells transfected with thy empty vector. This has been added to the caption.

Fig6: (A) Very poor-quality blots, there are bands from neighbouring lanes disturbing proper interpretation of the desired bands. It is also not clear which cell line was specifically used for in this blot.

Supplementary data has been added in this regard.

Fig6B: Legend should clearly state the parasites were incubated for 2h with host cells but not 48 h.
Methods: Assessment of invasion fitness should be performed after parasite removal (after 2h of parasite incubation). Further incubation of the cells for 48 h allows parasites to start intracellular replication leading to cofounding interpretations. Authors should elaborate why they have included this unusual step of 48 h incubation.

Figure caption has been modified for the sake of clarity.

Reviewer #3: The main goal of the manuscript is to describe the pathway by which cAMP/Epac participate in T.cruzi TCTs mechanism of host cell entry. Based on previous results from the group and the literature, the main hypothesis is that the pathway involves the activation of Rap1b, an effector downstream of Epac pathway.
The authors do show the ubiquitous activation of cAMp for different cell types. However, when they address the role of Rap1b in the cAMP/Epac pathway there are some important points in the data presented that need to be addressed and they are listed below:

1. The authors say in the text that “As shown in Figure 2, higher levels of activated GTP-bound Rap1 were detected in lysates from cells incubated with 8-Br-cAMP and trypomastigotes, supporting the involvement of Rap1b in cAMP-mediated invasion.” However statistically significant results are only observed for Hela cells exposed to T. cruzi Y strain or HL-1 treated with BrcAMP or exposed to Tc Y strain. The results for NRK cells are not statistically different from control cells. It is a fact that the bars do seem to show some differences, but the statistics did not say the same. So at least this sentence need to be rephrased.

Densitometry bands were adjusted and results slightly modified. Gel and densitometry and raw data can be found in as an attached file.

Later to prove the importance of Rap1b activation for T.cruzi host cell entry they used cells expressing a constitutively active form of Rap1b. However, they only use Hela and HL-1 for these experiments and no tests with NRKs are shown. If they really want to make a point that this pathway is ubiquitous for all cell lines, they need to perform these experiments also with NRKs. For these experiments they evaluate not only parasite cell invasion, but also the number of intracellular amastigotes and number of released parasites.

We agree that showing the mentioned results in NRK would have made a strong point regarding the pathway ubiquity. However, in our hands, even when using different reagents, the transfection of NRK cells has not efficient enough to have a reliable experiment.

This last one (# released parasites) though was only performed for HL-1 cells. Why? The same result was obtained for Hela? These data should be presented and discussed in the manuscript. In addition, although the number of released parasites may reflect the amount of cell infection/infected cells, there are a number of manuscripts in the field showing that invasion and intracellular multiplication are two different events and that released parasites are more related to the latter other than necessary to cell invasion. So authors need to be very careful to assume that number of released parasites are in fact a good measure of invasion levels.

Previous reported results for NRK (Musikant et al) and preliminary data for Hela, were similar to HL-1. HL-1, are cardiomyocytes from mouse heart, and heart one of the most important target organs in the infection and persistence of T. cruzi. For this reason and considering the fact that the pathway seems to be universal it was studied thoroughly only in HL-1 cells. We completely agree with the reviewer that invasion and intracellular multiplication are two different events and that released parasites are more related to the latter other than necessary to cell invasion.

2. In figure 4 the authors show a fluorescence microscopy assay, showing the co-localization of the parasite with Rap1b at the site of entry. How frequent this was observed? A quantification needs to be included in the figure. The assays were performed with HL-1 cells. Was this also observed for NRKs and Hela? Again in order to make an statement that this is an ubiquitous process for all types, this data should be included.

A quantification of colocalization has been added as suppl. figure 3. Results were also seen, but not shown, in Hela cells.

3. As mentioned before, invasion and intracellular multiplication are not necessarily related and there are data in the literature showing that parasite intracellular multiplication can be modulated by ROS, for example, so using parasite release is not a good method to test invasion. Thus, the results presented in figure 5, where authors test the influence of activated and not activated Rap1b in T. cruzi infection, should be performed using a classical invasion assay and not the number of released parasites. In this sense, the sentence “As shown in Figure 5, cells transfected with the phospho-mimetic Rap1b-S179D mutant presented a decreased invasion with respect to control cells or cells overexpressing the non-phosphorylable mutant Rap1b-S179A, supporting a PKA-dependent antagonistic effect on the pathway” is not what the results show. This correlation cannot be done and, as mentioned, in order to make this affirmative they need to perform an invasion assay showing the same results. It is possible that Rap1b may also interfere with parasite intracellular multiplication.

We agree with that choosing such an assay to show our hypothesis was not the best idea. However, previously reported results with PKA inhibitors and activators showed similar results than the ones presented here.

--------------------

PLOS authors have the option to publish the peer review history of their article ([what does this mean?](https://journals.plos.org/plosntds/s/editorial-and-peer-review-process#loc-peer-review-history)). If published, this will include your full peer review and any attached files.


If you choose “no”, your identity will remain anonymous but your review may still be made public.


**Do you want your identity to be public for this peer review?** For information about this choice, including consent withdrawal, please see our [Privacy Policy](https://www.plos.org/privacy-policy).

Reviewer #1: Yes: Carolina Cattoni Koh

Reviewer #2: No

Reviewer #3: No


Figure Files:
While revising your submission, please upload your figure files to the Preflight Analysis and Conversion Engine (PACE) digital diagnostic tool, https://pacev2.apexcovantage.com. PACE helps ensure that figures meet PLOS requirements. To use PACE, you must first register as a user. Then, login and navigate to the UPLOAD tab, where you will find detailed instructions on how to use the tool. If you encounter any issues or have any questions when using PACE, please email us at figures@plos.org.
 
Data Requirements:
Please note that, as a condition of publication, PLOS' data policy requires that you make available all data used to draw the conclusions outlined in your manuscript. Data must be deposited in an appropriate repository, included within the body of the manuscript, or uploaded as supporting information. This includes all numerical values that were used to generate graphs, histograms etc.. For an example see here: http://www.plosbiology.org/article/info%3Adoi%2F10.1371%2Fjournal.pbio.1001908#s5.
 
Reproducibility:
To enhance the reproducibility of your results, we recommend that you deposit your laboratory protocols in protocols.io, where a protocol can be assigned its own identifier (DOI) such that it can be cited independently in the future. Additionally, PLOS ONE offers an option to publish peer-reviewed clinical study protocols. Read more information on sharing protocols at https://plos.org/protocols?utm_medium=editorial-email&utm_source=authorletters&utm_campaign=protocols

__________________________________________________
In compliance with data protection regulations, you may request that we remove your personal registration details at any time. (Use the following URL: https://www.editorialmanager.com/pntd/login.asp?a=r). Please contact the publication office if you have any questions.
