# Supplementary material for: Host Cell Rap1b mediates cAMP-dependent invasion by Trypanosoma cruzi
Source: PLoS Negl Trop Dis. 2023 Mar 10;17(3):e0011191. doi: 10.1371/journal.pntd.0011191 (PMC10032529; doi:10.1371/journal.pntd.0011191)
Supplement: Attachment — Submitted filename: Response to reviewers R2.docx [file pntd.0011191.s008.docx]

Author’s Response to Reviewers

Once again, we would like to thank reviewers for taking the time to review our work and for the suggestions that have definitively improved our manuscript.

**Methods**


-Are the objectives of the study clearly articulated with a clear testable hypothesis stated?

-Is the study design appropriate to address the stated objectives?

-Is the population clearly described and appropriate for the hypothesis being tested?

-Is the sample size sufficient to ensure adequate power to address the hypothesis being tested?

-Were correct statistical analysis used to support conclusions?

-Are there concerns about ethical or regulatory requirements being met?


Reviewer #1: Describe the concentration of DMSO used (line 179).

DMSO concentration has been added in materials and methods section.

Reviewer #3: no new comments

--------------------

**Results**


-Does the analysis presented match the analysis plan?

-Are the results clearly and completely presented?

-Are the figures (Tables, Images) of sufficient quality for clarity?


Reviewer #1: no comments

Reviewer #3: There are some points that still need to be addressed and will be listed in the general comments

--------------------

**Conclusions**


-Are the conclusions supported by the data presented?

-Are the limitations of analysis clearly described?

-Do the authors discuss how these data can be helpful to advance our understanding of the topic under study?

-Is public health relevance addressed?


Reviewer #1: no comments

Reviewer #3: There are some points that still need to be addressed and will be listed in the general comments

--------------------

**Editorial and Data Presentation Modifications?**


Use this section for editorial suggestions as well as relatively minor modifications of existing data that would enhance clarity. If the only modifications needed are minor and/or editorial, you may wish to recommend “Minor Revision” or “Accept”.

Reviewer #1: On the Y axes of the figures it is written: normalized or relative to something . For me, the analysis and meaning of both is the same. I suggest that it be standardized.
Y axes has been standardized.

Reviewer #3: (No Response)

--------------------

**Summary and General Comments**


Use this section to provide overall comments, discuss strengths/weaknesses of the study, novelty, significance, general execution and scholarship. You may also include additional comments for the author, including concerns about dual publication, research ethics, or publication ethics. If requesting major revision, please articulate the new experiments that are needed.

Reviewer #1: no comments

Reviewer #3: The authors have significantly improved the manuscript, addressing the points raised in the first review. However, there are still some relevant points to be addressed.

-In figure 3, again, after 48h you already have parasite multiplication. To attest that this is related to invasion, they need to analyze the cells right after parasite exposure. Also, it is not clear what they are calling invasion. Is it the number of infected cells? This should be clearer in the methods and in the legend of the figure. It would be important, since in the way they presented the results (relative to non-transfected) we cannot have an idea of the number of parasites/cell, which would indicate whether RAP1b does interfere with parasite multiplication. This clarification and discussion is important, since the results could still be related to parasite multiplication, rather than only invasion. Multiplication alone could account for more parasites being released from the culture.

Sensitivity of the assay makes too hard to count parasites immediately after exposure. For this reason, an infection model consisting in a 2h incubation of parasites with host cells and another 48h incubation without free parasites, was originally set. It is true that there is replication after 48hs and, in the case of transfected cells, it is not possible to exclude an effect of the constitutive active Rap1b. A discussion of this fact has been added in the discussion section.

In agreement with the reviewer, it is not invasion but infection what we are evaluating in transfected cells, so, for the sake of clarity, “invasion” has been replaced by the term “infection”.

- Still concerning Figure 3, as a response to a comment from the first review the authors mentioned they used only HL-1 for parasite release, because it is a cardiomyoblast, a cell type important for Chagas disease. Also, because this had already been shown previously for NRKs.
It should be also mentioned in the text that there is already published data showing similar results for NRKs and also include a supplementary figure showing the preliminary data for Hela. This is important to prove the point that this pathway is in fact ubiquitous. With the results shown, they can only speculate that, due to some data in the literature, as well as preliminary results, this may be an ubiquitous pathway.

In figure 3, results presented correspond to HL-1 and Hela. We believe reviewer is talking about figure 5, in which only results obtained for HL-1 are presented. As mentioned, our previous work using PKA inhibitors in invasion assays on NRK cells put in evidence a potential PKA-Epac cross-talk. In this work, inhibition of PKA induced an increase of invasion (Musikant D, et al. Host Epac1 is required for cAMP-mediated invasion by Trypanosoma cruzi. Molecular and Biochemical Parasitology. 2017;211:67–70)), equivalent of what we see in this work. Since in our hands NRK transfection has not been efficient, we were not been able to repeat the assay presented here. Results in Hela are too preliminary to be incorporated. For these reasons, we have modified the text as suggested by the reviewer.

-Concerning figure 4, results with Hela should be mentioned in the text and included as a new supplementary figure. This is important to strengthen the point that this is a ubiquitous process. Otherwise, the authors should revise this statement and say that they observed it only for HL-1 and that, according to preliminary data, it might work the same way for other cell types. Supplementary figure 4 should be included as a new panel in this figure.

Hela results have been mentioned in the text and additional data added to supplementary figure 4.

-In response to the comment made to figure 5 and the affirmative that due to more released parasites, the invasion rates were higher. The authors replied that this statement was valid, since “previously reported results with PKA inhibitors and activators showed similar results”. This is not the case. In order to make that affirmative “there was more invasion” it is needed to be shown, that there are more internalized parasites/ infected cells or even more cells infected at time 0, right after parasite exposure. They cannot use “more invasion” if what they are showing is “more parasite release”. The fact that there are data in the field that could corroborate this idea, does not allow them to assume this is true, they could only speculate that this might be the case and include the mentioned reference. This must be changed in the text and the legend of the figure, as well as in the discussion of the data.
Text has been changed in accordance to reviewer’s suggestions.

--------------------

PLOS authors have the option to publish the peer review history of their article ([what does this mean?](https://journals.plos.org/plosntds/s/editorial-and-peer-review-process#loc-peer-review-history)). If published, this will include your full peer review and any attached files.


If you choose “no”, your identity will remain anonymous but your review may still be made public.


**Do you want your identity to be public for this peer review?** For information about this choice, including consent withdrawal, please see our [Privacy Policy](https://www.plos.org/privacy-policy).

Reviewer #1: No

Reviewer #3: No


Figure Files:
While revising your submission, please upload your figure files to the Preflight Analysis and Conversion Engine (PACE) digital diagnostic tool, https://pacev2.apexcovantage.com. PACE helps ensure that figures meet PLOS requirements. To use PACE, you must first register as a user. Then, login and navigate to the UPLOAD tab, where you will find detailed instructions on how to use the tool. If you encounter any issues or have any questions when using PACE, please email us at figures@plos.org.
 
Data Requirements:
Please note that, as a condition of publication, PLOS' data policy requires that you make available all data used to draw the conclusions outlined in your manuscript. Data must be deposited in an appropriate repository, included within the body of the manuscript, or uploaded as supporting information. This includes all numerical values that were used to generate graphs, histograms etc.. For an example see here: http://www.plosbiology.org/article/info%3Adoi%2F10.1371%2Fjournal.pbio.1001908#s5.
 
Reproducibility:
To enhance the reproducibility of your results, we recommend that you deposit your laboratory protocols in protocols.io, where a protocol can be assigned its own identifier (DOI) such that it can be cited independently in the future. Additionally, PLOS ONE offers an option to publish peer-reviewed clinical study protocols. Read more information on sharing protocols at https://plos.org/protocols?utm_medium=editorial-email&utm_source=authorletters&utm_campaign=protocols

__________________________________________________
In compliance with data protection regulations, you may request that we remove your personal registration details at any time. (Use the following URL: https://www.editorialmanager.com/pntd/login.asp?a=r). Please contact the publication office if you have any questions.
